# Enhanced Precision of Fluorescence In Situ Hybridization (FISH) Analysis Using Neural Network-Based Nuclear Segmentation for Digital Microscopy Samples

**DOI:** 10.3390/s26030873

**Published:** 2026-01-28

**Authors:** Annamaria Csizmadia, Bela Molnar, Marianna Dimitrova Kucarov, Krisztian Koos, Robert Paulik, Dora Kapczar, Laszlo Krenacs, Balazs Csernus, Gergo Papp, Tibor Krenacs

**Affiliations:** 1 Doctoral School of Pathological Sciences, Semmelweis University, H-1085 Budapest, Hungary; csizmadia.annamaria@phd.semmelweis.hu; 2 3DHISTECH Ltd., H-1141 Budapest, Hungary; bela.molnar@3dhistech.com (B.M.);; 3 Department of Internal Medicine and Oncology, Semmelweis University, H-1083 Budapest, Hungary; 4 Doctoral School of Applied Informatics and Applied Mathematics BioTech, Obuda University, H-1034 Budapest, Hungary; kucarov.marianna@uni-obuda.hu; 5 Synthetic and Systems Biology Unit, Biological Research Center, H-6726 Szeged, Hungary; krisztian.koos@gehealthcare.com; 6 Department of Pathology and Experimental Cancer Research, Semmelweis University, Üllői út 26, H-1085 Budapest, Hungary; dora.kapczar@semmelweis.hu (D.K.); papp.gergo@semmelweis.hu (G.P.); 7 T-Cell Ltd., Laboratory of Tumor Pathology and Molecular Diagnostics, H-6726 Szeged, Hungary; krenacsl@vipmail.hu

**Keywords:** nuclear segmentation, overlapping nuclei, follicular lymphoma, fluorescence in situ hybridization, FISHQuant, Cellpose, NucleAIzer, StarDist

## Abstract

**Introduction:** Accurate nuclear segmentation is essential for the reliable diagnostic interpretation of fluorescence in situ hybridization (FISH) results. However, automated 2D digital algorithms often fail in samples with dense or overlapping nuclei, such as lymphomas, due to the loss of spatial depth information. Here, we tested if AI-based 3D nuclear segmentation can improve the accuracy, reproducibility, and diagnostic reliability of FISH reading in critical situations. **Materials and Methods:** Formalin-fixed follicular lymphoma sections were FISH-labeled for BCL2 gene rearrangements and digitally scanned in multilayer Z-stacks. The analytic performance in nuclear segmentation of the adaptive thresholding-based FISHQuant, and the freely accessible AI-based NucleAIzer, StarDist, and Cellpose algorithms, were compared to the eye control-based traditional FISH testing, primarily focusing on nuclear segmentation. **Results:** We revealed that the Cellpose algorithm showed limited sensitivity to low-intensity signals and the adaptive thresholding 2D segmentation, and FISHQuant struggled to resolve densely packed nuclei, occasionally underestimating their counts. In contrast, 3D segmentation across focal planes significantly improved the nuclear separation and signal localization. AI-driven 3D models, especially NucleAIzer and StarDist, showed improved precision, lower variance (VP/VS ≈ 0.96), and improved gene spot correlation (r > 0.82) across multiple focal planes. The similar average number of gene spots per cell nuclei in the AI-based analyses as the eye control counting, despite the elevated number of cell nuclei found with AI, validated the AI nuclear segmentation results. **Conclusions:** Inaccurate segmentation limits automated diagnostic FISH signal evaluation. Deep learning 3D approaches, particularly NucleAIzer and StarDist, may overcome thresholding and 2D constraints and improve the consistency of nuclear detection, resulting in better classification of pathogenetic gene aberrations with automated workflows in digital pathology.

## 1. Introduction

**Figure 1:**
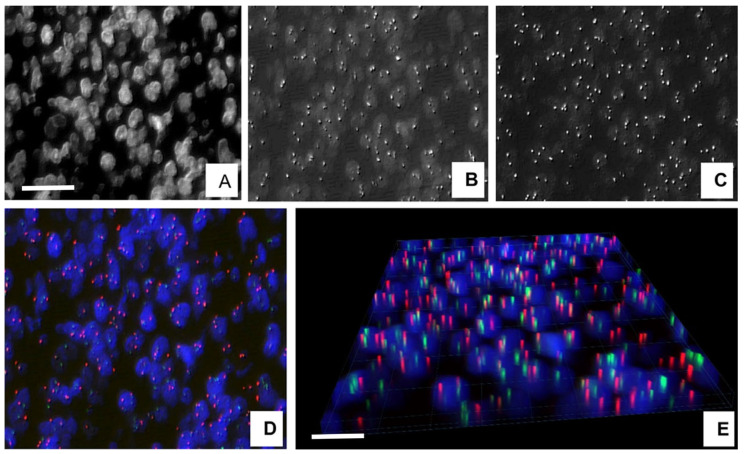
Three-dimensional visualization of digitalized FISH images comprising 7 focal planes (z-layers) of 0.4 µm spacing in follicular lymphoma. The projection of specific gene signals within the spatial nuclei is clearly demonstrated across the whole section thickness. DIC (Digital Differential Interface Contrast) images of nuclei (**A**), with gene signals of 14q32 (**B**) and 18q22 (**C**). Colored DIC image of combined 14q32 (FITC—green) and 18q22 (TRITC—red) signals. Cell nuclei are blue (AMCA) (**D**) as visualized in 3D (**E**). Scale bare on A represents 25 µm in (**A**–**D**), on (**E**) = 15 µm.

Fluorescence in situ hybridization (FISH) can identify specific genetic aberrations in cell specimens and tissue sections for differential diagnosis and for confirming potential targets of therapy in malignant tumors [1]. For relevant results, the number, proportion, spatial arrangement, and distance from each other of <0.5 µm diameter fluorescing signals of two or more colors must be precisely analyzed within individual cell nuclei. This can usually be achieved without difficulties in tumor cells of decent cytoplasm, which keep apart adjacent cell nuclei, but it is complicated, e.g., in lymphomas with touching or overlapping nuclei in a ~4–5 µm thick tissue section, either using eye-controlled or machine learning-based automated FISH analysis [2,3], as it can be demonstrated in a follicular lymphoma (FL) (Figure 1). Therefore, improving the accuracy of automated digital separation of cell nuclei in critical samples such as lymphomas can essentially influence the precision of specific FISH signal patterns and the diagnostic conclusion drawn.

Despite the fact that next-generation digital fluorescence microscopy supports multi-planar image acquisition providing detailed 3D volumetric information, the 2D nuclear segmentation methods are still widely used in relevant workflows [4]. Their computational processing speed, efficiency, simplicity, and compatibility with established pipelines render them particularly suitable for high-throughput diagnostic settings. On the other hand, 3D imaging produces extensive datasets, processing which requires advanced computational infrastructure, storage capacity, and retrained personnel [3]. However, 2D nuclear segmentation is inherently limited by the loss of depth information, which restricts its ability to capture spatial relationships between structures across focal planes. This becomes particularly evident when analyzing complex samples with overlapping or densely packed nuclei, where the absence of contextual depth reduces segmentation accuracy [5]. 

The cornerstone of cell-based automated image analysis workflows is the reliable segmentation of cell nuclei in complex microscopy samples, for which several algorithms using diverse approaches have been published [4,6]. Our group has developed and partly upgraded semi-automated algorithms within the FISHQuant package for FISH analyses of the most frequent genetic aberration types, i.e., copy number alterations and gene rearrangements/translocations based on 2D nuclear segmentation [7]. The software package proved to be useful in supporting an effective workflow management and remote diagnostics with similar results compared to eye-controlled signal counting [8]. 

Novel deep learning-based nuclear segmentation algorithms offered open-source, generalized solutions for the reliable segmentation of individual cell nuclei in microscopy images. Some of these algorithms are advertised to eliminate the need for additional training, allowing immediate applicability across different imaging modalities. 

NucleAIzer is a neural network-based algorithm that employs automated nucleus-style model adaptation through an image style transfer framework [9]. It can support expert-annotation-free segmentation of 2D and 3D cell nuclei in both light and fluorescence microscopy images. Accessible online (at www.nucleaizer.org, accessed on 20 October 2025) (http://www.nucleaizer.org, accessed on 20 October 2025) and integrated into the CellProfiler software suite, NucleAIzer demonstrated exceptional performance, achieving top scores in the 2018 Data Science Bowl competition among 739 participants [9].

The Cellpose program (https://github.com/MouseLand/cellpose, accessed on 20 October 2025), pre-trained on over 70.000 diverse fluorescence cellular objects, enables 3D nuclear segmentation by utilizing two-dimensional (2D) model data [10]. It employs a gradient prediction algorithm across individual 2D planes in the xy, xz, and yz dimensions. Due to the extensive and diverse pre-trained datasets, it demonstrated robust performance in identifying nuclei with complex morphologies, even in previously unseen sample types [11]. Additionally, retraining Cellpose on user-specific data can further enhance its predictive accuracy and adaptability.

The StarDist program is capable of training neural networks on user datasets, utilizing star-convex polygons for the precise segmentation of cell nuclei [12,13]. The algorithm was created to overcome the limitations of others in handling blurry, crowded, or atypical 2D images. Its version adapted to 3D image stacks was developed to manage densely clustered cells and nuclei within image stacks, even in the presence of significant background noise [3]. The enhanced precision of 3D over the 2D nuclear segmentation in testing complex structures can be demonstrated by comparing the results of different algorithms applied in the same area of a follicular lymphoma sample after LSI BCL2 Break-Apart Dual-Color and LSI BCL2/IGH Dual-Color FISH reaction. 

The eye control-based method was chosen as a gold standard, since most diagnostic laboratories still use traditional FISH analysis of at least 50–200 tumor cells per sample, depending on the type of tumor and gene aberration required by the guidelines, for a reliable diagnostic decision making [14,15]. This fact and the frequent overlapping nuclei in lymphoma sections emphasized the need to search for automation. We compared the analytic performance of FISHQuant and the AI-based, general-purpose Cellpose, NucleAIzer, and StarDist algorithms in order to find which can improve the accuracy and reproducibility of FISH reading and reduce the chance of bias and the fatigue of pathologists.

In this paper, the accuracy and reproducibility of digital 2D and 3D nuclear segmentation algorithms were tested and compared to that of the eye-controlled FISH analysis results using multilayer digitalization of follicular lymphoma samples of crowded, overlapping tumor cell groups. The majority of follicular lymphomas is featured by the t(14;18)(q32;q21) translocation, where the BCL2 gene from chromosome 18 is rearranged next to the IgH promoter on chromosome 14, resulting in the upregulation of the anti-apoptotic BCL2 protein, which drives the autonomous growth of lymphoma cells [16,17,18,19]. For confirming BCL2 translocation and upregulation, both the SPEC BCL2 Dual-Color Break-Apart Probe and the SPEC BCL2/IGH Dual-Color Dual-Fusion FISH probes were applied in a series of FL sections for assessing the comparative performance of the listed nuclear segmentation algorithms. Our primary endpoint was the per-case F1-score for correct genetic classification (fusion, break-apart, normal), while all segmentation-level metrics were treated as secondary endpoints.

## 2. Materials and Methods

### 2.1. Fluorescence In Situ Hybridization (FISH) Method

Formalin-fixed and paraffin-embedded (FFPE) tissue samples from the routine files of the Tumor Pathology and Molecular Diagnostic Laboratory (Szeged) were used for FISH analysis in this study, in line with the regulations of the WMA Declaration of Helsinki. This research was approved by the National Committee for Research Ethics (ETT TUKEB) under the permission No. BMEÜ/443-5/2022/EKU issued in 10/10/22. The Committee waived the need for individual patient consent on archived, diagnosed tissues for the purpose of retrospective biomarker testing. 

**Figure 2:**
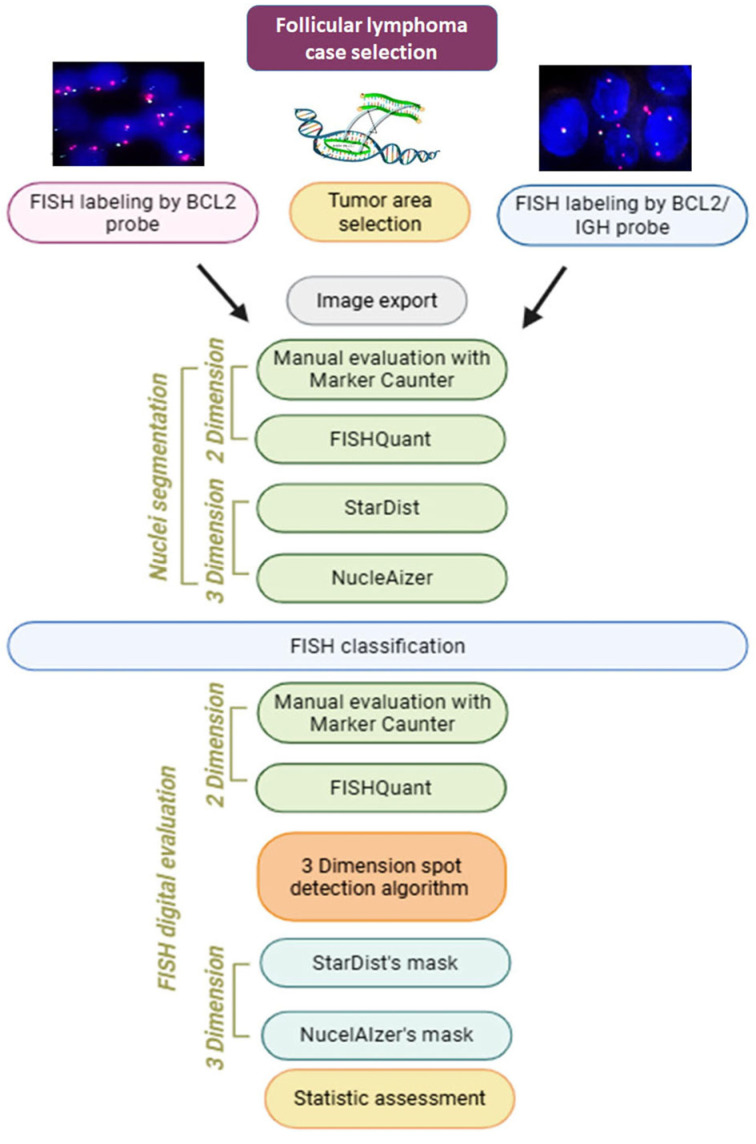
Study design. Consecutive tissue sections of follicular lymphoma cases, marked either with LSI BCL2/IGH DC DF or BCL2 Break-Apart probes, were digitalized and representative tumor areas were analyzed. The results of eye-controlled counting of cell nuclei and gene spots utilizing the MarkerCounter software was then compared to those gained using the FISHQuant and the AI-based (initially using Cellpose too), StarDist, and NucleAIzer algorithms. The orientation of signals within cell nuclei, identified with AI algorithms, was determined using a signal decoding algorithm for each digital layer of the sections.

Lymph node sections from 5 follicular lymphoma (FL) cases were used for confirming the t(14;18)(q32;q21) chromosomal translocation, resulting in the upregulation of BCL2 protein expression, which were tested. Figure 2 summarizes our digital FISH analysis workflow in this study. For FISH, both the SPEC BCL2 Dual-Color Break-Apart Probe and the SPEC BCL2/IGH Dual-Color Dual-Fusion Probe were applied in consecutive tissue sections (all Zytolight; Pentagen, Kladno, Czech Republic). Briefly, the FISH reactions involved the following steps: routine dewaxing of paraffin-embedded tissue sections (3 × 30 min in xylene; then 3 × 30 min in abs. ethanol), washing 2 × 3 min in 2× SSC, boiling for 15’ in a pH 6.0 citrate buffer. and 15 min cooling to room temperature. Then, washing in 2× SSC at 37 °C, digestion for 15 min using 10% pepsin (Sigma-Aldrich, P6887) at 37 °C, washing in 2× SSC, then dehydration (in graded 70-80-100% ethanol series), brief drying and applying 6 µl FISH probe, followed by 10 min denaturation at 85 °C, then overnight hybridization at 37 °C. Finally, post-hybridization washing of the samples in 0.4× SSC at 70 °C and then in 2× SSC containing 0.1% NP-40 detergent for 5 min each was followed by slide mounting using a medium containing DAPI (4’,6-diamino-2-phenylindole) to reveal cell nuclei.

### 2.2. Digital Imaging and Image Processing 

Whole slide digitalization of FISH reactions was performed in a CE-compliant (98/79/EC in vitro diagnostic directive) Pannoramic MIDI II fluorescence scanner (3DHISTECH Ltd., Budapest, Hungary) using a x40 NA:0.8 Plan Apochromat objective and a PCO edge 4.2 bi USB SCMOS camera. Appropriate fluorescence scanning profiles were adapted to the DAPI (Ex:358/50nm-Em:405/80nm), Spectrum Green (Ex:497/30nm-Em:538/44nm), and Spectrum Orange (Ex:543/22nm-Em:586/20nm) filters, respectively, in z-layer scanning mode, including 7 layers with 0.4 µm distance between each.

Manual annotation was performed on multilayer scanned FISH images (which approach is close to 3D) by 2 pathologists based on images of consecutive H&E-stained slides, which allowed testing for BCL2 gene fusion/translocation labeling on representative tumor areas. ROI (region of interest) areas were selected by hematopathologists (LK and BT) based in follicle-like structures of frequent overlapping nuclei to include at least 1000 cells each, excluding areas of autofluorescing red blood cells. Digital FISH slides were independently reviewed by two board-certified hematopathologists who were blinded to the algorithmic outputs. Inter-rater agreement was quantified using Cohen’s kappa statistics. To adjudicate cases with overlapping or densely packed nuclei, a blinded micro-panel of three additional pathologists was convened. This panel evaluated a representative subset of nuclei where AI-based segmentation suggested nuclear splits, and reached a consensus on whether the proposed separations were biologically plausible. 

The same areas of digital slides FISH—labeled on consecutive sections either using the BCL2 Dual-Color Dual-Fusion or Break-Apart probes—were analyzed. Cell nuclei and FISH spot segmentation results obtained using the IVDD-qualified 2D FISHQuant image analysis software package of the QuantCenter image analysis platform (3DHISTECH Ltd.) were compared to those generated by the AI-based image processing methods StarDist (version 0.7.0), NucleAIzer, and Cellpose (version 1.0) [https://www.cellpose.org/, accessed on 20 October 2025]. The PANNORAMIC^®^ CaseViewer (version 2.5 RTM) slide alignment tool enabled the preselected tumor annotations to occupy identical positions across consecutive tissue sections labeled with different FISH probes. This ensures consistent evaluation across the image processing algorithms. The identified study areas were exported in Tile TIFF file format as grayscale images and the Image J program 1.53v (NIH https://imagej.net/ij/download.html, accessed on 20 October 2025) was used for counting the detected nuclei by AI-based image processing algorithms. 

### 2.3. Manual Counting of Cell Nuclei and Gene Spot in Digital Slides

For manual evaluation of the digital slides, the MarkerCounter function of the Pannoramic Viewer software was utilized. This tool uses a single-layer format of digital slides, integrating all focal planes into a unified extended focus image. This approach is very useful for counting objects in tissue sections of variable thickness. The extended focus image selects high-intensity pixels of individual color channels of all focal planes and combines them into a two-dimensional composite image. Cell nuclei were manually annotated and their number summed up, which allowed precise data extraction and analysis.

### 2.4. Digital Segmentation of Cell Nuclei and Gene Spots

#### 2.4.1. FISHQuant Adaptive Threshold Algorithm 

FISHQuant dynamically examines and adjusts local intensity threshold values to segment cell nuclei in FISH images [8]. It utilizes RGB-transformed grayscale images to maintain robust segmentation under varying imaging conditions. The adaptive thresholding divides images into smaller local regions, known as blocks or windows, which are analyzed separately, based on their local intensity characteristics, to compensate for fluorescence intensity fluctuations. Then, grayscale images are transformed into a binary mask, where the segmented nuclei appear as distinct regions. Regional thresholding is based on local mean, as a Gaussian-weighted mean, which assigns more significance to pixels near the object centers. If the pixel intensity is higher than the threshold, the pixel is set to white, whereas if it is lower, it is set to black. Additional filtering, such as noise removal and edge smoothing, as well as watershed-based nuclear separation when nuclei overlap enhances the accuracy of nuclear segmentation, is implemented. The drawback of adaptive thresholding can be linked to computational demand/cost and its semi-automated nature due to regional style analyses. 

#### 2.4.2. AI-Based Cell Nuclei Detection

For automated segmentation of nuclei in FISH images, we utilized AI-based image analysis algorithms, including StarDist, NucleAIzer, and Cellpose, alongside the adaptive thresholding-based FISHQuant method. All these were applied to grayscale images obtained from multiple Z-stack layers to test nuclear segmentation in follicular lymphoma (FL) tissue samples. StarDist employs a convolutional neural network (CNN) that models nuclei as star-convex polygons, ensuring high accuracy in segmenting round or elliptical nuclei with well-defined boundaries [12,13]. Its ray-based encoding approach enables precise delineation of individual cell nuclei, even in densely packed or overlapping regions. NucleAIzer (www.nucleaizer.org, accessed on 20 October 2025), developed for the Kaggle 2018 Data Science Bowl, incorporates a Mask R-CNN-based segmentation framework with U-Net-based boundary refinement, augmented by style transfer techniques to improve generalizability across different microscopy image datasets [9].

Cellpose was originally pre-trained on over 70.000 diverse fluorescence cellular objects, showed robust performance, and it was suggested to work in previously untrained samples. It is a shape-flow modeling-based approach which uses a general-purpose segmentation strategy; however, it has a suggested vulnerable stability to varying intensity fluorescence [20,21]. To mitigate challenges associated with focal plane selection in traditional 2D imaging, here we employed extended focus imaging, resulted from the merging of multilayer sample scanning. Tested areas digitized at different focal depths were processed by selecting the highest-intensity pixels in individual color channels, ensuring that the final image preserved high-quality structural details. All free-access, general-purpose AI tools were used off-the-shelf, without any further training method.

#### 2.4.3. Automated Gene Spots Detection

The algorithm detecting gene-specific fluorescence signal spots in situ within cell nuclei can reveal genetic aberrations of diagnostic and therapeutic potential. The primary processing steps detect fluorescing FITC (green) and TRITC (red) spots in the initial phase DAPI (blue) masks, which highlight all nuclear deoxyribonucleic acids (DNAs) to effectively identify cell nuclei in tissue samples. DAPI masks undergo grayscaling, noise reduction, and binarization, followed by a segmentation step defined using parameters such as contour hierarchy, area, and center. When these parameters align, the potential contours of cell nuclei are assessed to determine if previously registered nuclear center points lie inside, outside, or along the edge of the new contour. If all center points are outside the new contour center, along with a unique nucleus ID, data are registered in a global nuclei ID-to-nucleus center dictionary, ensuring a non-redundant dictionary for all scanned FITC-TRITC images. Auto nuclei ID registration algorithm enables avoiding the evaluation of the same nuclei more than once. 

In the second phase (green in Figure 2), scanned FITC-TRITC signals under the same annotated areas are preprocessed using channel splitting into R, G, B, grayscaling, and blurring in parallel with DAPI processing. In the third phase (orange in Figure 2), the results of the first two phases are combined. The peak pixel intensity values in the grayscaled FITC and TRITC scanned images mark the potential gene spot centers within the contours of cell nuclei registered through the DAPI masks. Then, the coordinates of spot centers are extended around to include pixels above a preselected intensity threshold for defining the spot‘s total area. Any prior spots are assigned for the same nucleus ID. If there is no overlap, the new spot center is registered in a global nucleus ID-to-spot centers dictionary. This non-redundant dictionary facilitates spot visualization within the colored FITC/TRITC scans. 

### 2.5. Statistical Analysis 

Pearson correlation testing was employed to evaluate the consistency of nuclei detection between AI-based segmentation algorithms and the adaptive thresholding method in FISHQuant. A high correlation indicates strong agreement, while a lower correlation suggests the supremacy of AI-based segmentation. In the evaluation of AI-driven and conventional segmentation techniques, Sample Variance (VS) and Population Variance (VP) served as critical statistical measures for assessing accuracy and consistency [22] (VS quantifies dispersion within a sample as a key indicator of segmentation reliability across multiple images. Low VS suggests consistent nuclei detection and algorithmic robustness across varying scenarios (e.g., image quality or illumination) of AI-based segmentation over adaptive thresholding. High variance values indicate a need for further optimization. VP, on the other hand, measures overall dataset variability, reflecting the adaptability of the algorithm to diverse biological samples, imaging conditions and tissue types. By analyzing VS and VP, we could systematically assess the reliability and generalizability of AI-based nuclei segmentation methods, ensuring their suitability for complex biological image analysis. In this study, the primary endpoint was the per-case F1-score for correct genetic classification (fusion, break-apart, normal), while all segmentation-level metrics were treated as secondary endpoints. Key evaluation metrics including Recall, Precision, F-measure, and Accuracy also offer insights into algorithm performance. Recall quantifies the algorithm’s ability to detect relevant nuclei, defined as the ratio of correctly identified nuclei to the total actual nuclei. Precision measures the proportion of correctly identified nuclei among all detections, reflecting the algorithm’s effectiveness in minimizing false positive objects. The F-measure (F1-score) balances Recall and Precision, providing a single metric that accounts both for false positives and false negatives. It is particularly valuable in AI-based segmentations, where optimizing one metric often impacts the other. Accuracy assesses overall algorithmic correctness by measuring the proportion of correctly classify objects (true positives and true negatives) among all predictions. 

## 3. Results

### 3.1. Comparing the Efficiency of Cell Nuclei Segmentation Algorithms

**Figure 3:**
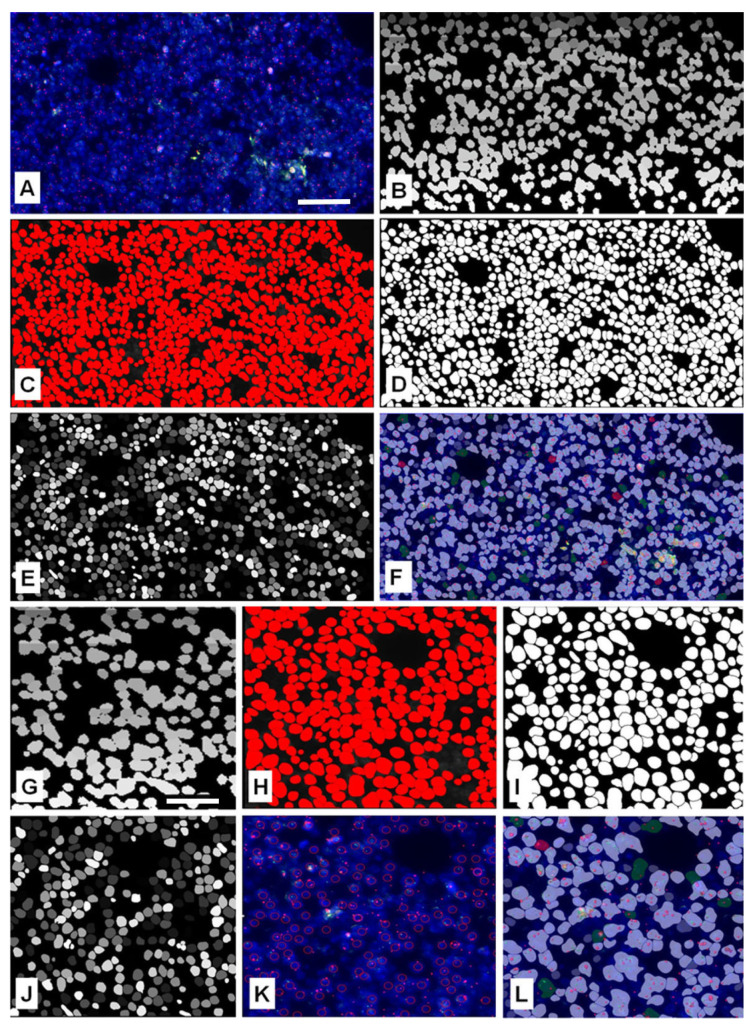
Cell nuclei segmentation in the same annotated area of a follicular lymphoma after FISH labeling using a Dual-Color Break-Apart probe for the detection of the t(14;18)(q32;q21) gene translocation (**A**). Cell nuclei are highlighted after testing with the Cellpose (**B**), StarDist (**C**), greyscale StarDist (**D**), NucleAIzer (**E**), or FISHQuant (**F**) algorithms. High-power views (10×) of similar lymph node areas more clearly show details of cell nuclei separation by the Cellpose (**G**), StarDist (**H**), greyscale StarDist (**H**,**I**), NucleAIzer (**J**), MarkerCounter (**K**), and FISHQuant methods (**L**). Gene spots in red and green can also be seen on A, F, K and L. Scale bar on A represents 50 µm on (**A**–**F**); scale bar on G represents 20 µm on (**G**–**L**).

The kappa correlation between two pathologists on the detected number of cell nuclei using eye-controlled examination with MarkerCounter was high (κ = 0.93186). Overall, the narrow ranges and moderate deviations confirm that the manual quantification was reproducible, with no evidence of extreme discrepancies or systematic bias. We tested the precision of automated nuclear and gene signal segmentation algorithms in digital slides of follicular lymphomas featured by crowded overlapping tumor cell nuclei following FISH, either using BCL2 gene Dual-Fusion or Break-Apart FISH probes. Only the obviously separated cell nuclei were annotated and counted under eye control when using the MarkerCounter algorithm of Pannoramic Viewer, the data which served as the gold standard in assessing the segmentation performance of the automated image analysis platforms. Visual examples of the nuclear separation results by the adaptive thresholding-based FISHQuant and the AI-based NucleAIzer, Cellpose, and StarDist algorithms in the same FL sample are shown in Figure 3. Most of the currently available image analysis tools perform automated processing based primarily on single-plane 2D images, typically corresponding to the microscope’s focal plane. Although more advanced techniques such as extended focus imaging (EFI), which integrates information from multiple focal planes, can offer a more comprehensive spatial representation, these are not yet widely implemented into standard workflows [23]. While the FISHQuant algorithm utilizes extended focus images, the AI-based image processing tools were evaluated both on the 0th optimal focal planes for the 2D segmentation tests and the Z-stack digitized images for assessing their 3D performance.

**Figure 4:**
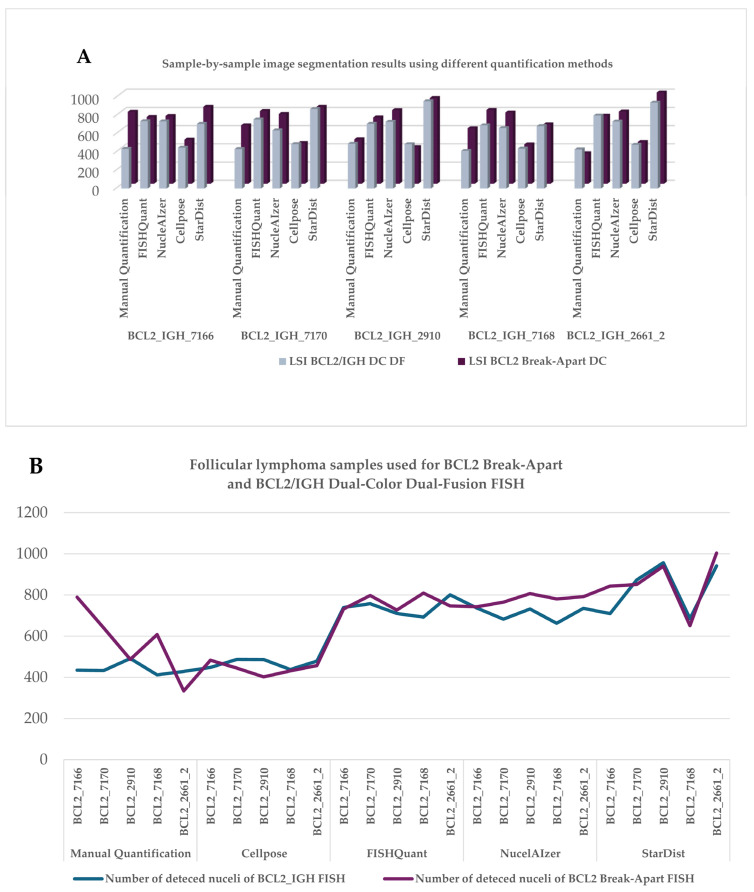
Counting of DAPI-stained cell nuclei within standard annotations of follicular lymphoma samples using eye control, adaptive threshold-based FISHQuant, and machine learning algorithms including Cellpose, NucleAIzer, and StarDist after BCL2 Break-Apart and BCL2/IGH gene fusion FISH reactions on single-layer images (**A**). Comparison of different image processing algorithms on follicular lymphoma cases FISH-labeled using either BCL2 Dual-Color Break-Apart or BCL2/IGH DC DF probes. The number of detected cell nuclei by each method in each case grouped by the algorithms used (**B**).

Initially, we evaluated the 2D nuclear segmentation capabilities of the AI-based algorithms. The results demonstrated that all nuclear segmentation/separation methods identified substantially more nuclei compared to the eye controlled manual evaluation using MarkerCounter. Among the AI-powered image segmentation tools, NucleAIzer and StarDist detected significantly more nuclei, by clearly outperforming both the eye-control-based and semi-automated image processing (FISHQuant) approaches (Figure 4A,B). However, the general-purpose AI-based Cellpose software markedly underperformed compared to the other tested algorithms. As expected, the different FISH labeling techniques had no major impact on the performance of any of the nuclear segmentation tools.

The Pearson coefficient (r = 0.832525) indicated a strong correlation between the number of nuclei identified by the tested image segmentation algorithms when comparing the consecutive sections labeled with either of the two FISH probes. The Pearson analysis revealed varying degrees of correlation among the performance of the segmentation algorithms. For instance, while the correlation between the StarDist and Cellpose algorithms was generally weak, the latter showed good associations with other methods. Overall, the eye-controlled evaluations resulted in moderate correlation with data gained using either the Cellpose, NucleAIzer, or StarDist algorithms (Table 1). The negative Pearson correlation indicated that the given methods detected fewer nuclei than others. This inverse relationship likely stems from fundamental differences in how the algorithms interpret nuclear size, shape, or intensity data. This underscores their different sensitivities, affecting both quantitative reliability and influence method selection. In most cases, the NucleAIzer method consistently segmented significantly higher number of nuclei than all other nucleus detection approaches. Strong inverse correlations were observed between NucleAIzer and manual evaluation, FISHQuant, and Cellpose, as well as between StarDist and FISHQuant methods (Table 1). Cellpose was excluded from further 3D analysis due to its consistently low performance in initial 2D tests and failure to detect low-intensity signals.

### 3.2. Multilayer Segmentation of Cell Nuclei

**Figure 5:**
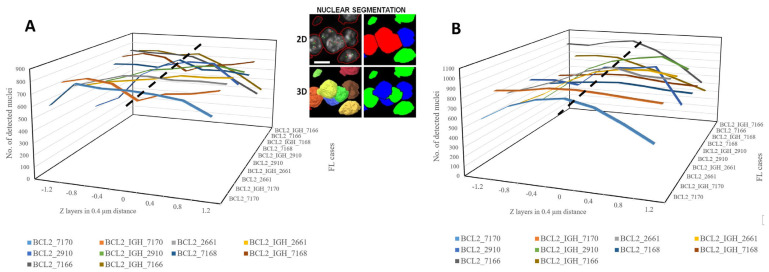
The number of cell nuclei identified in follicular lymphomas (FL) using either the NucleAIzer (**A**) or the StarDist (**B**) deep learning-based nuclear segmentation algorithms across all focal planes in all cases. Three-dimensional segmentation and reconstruction of cell nuclei contours may allow more accurate nuclei separation compared to 2D analysis, as demonstrated in the same cell cluster on the middle panel. Scale bar on middle figure represents 5 µm in all images.

Besides the 2D extended focus image analysis, the nuclear contours in FISH samples were also tested across all recorded digital layers for 3D reconstruction using the robust AI-based nuclear segmentation algorithms NucleAIzer and StarDist. During multilayer digitization of tissue sections, the focus plane—defined as that containing the highest-intensity pixel values within the specified fluorescence channel—was determined first. Then, the Z-axis stepping motor of the digital microscope moved up and downward from this zero-plane focus position without further refocusing. Each FISH section was digitized in seven planes with a 0.4-micron step size in Z directions. The nuclear contours slightly changed as the focal planes moved away from the zero point into both directions. Pixel intensity values decreased significantly below and above the 0 plane in the sample. Therefore, nuclear contours and counts were the highest in the focal planes and decreased by the distance from it (up and down) (Figure 5A,B), which occasionally allowed more precise cell nuclei separation of FISH-stained cell nuclei, as seen in middle images on Figure 5.

### 3.3. Series Measurement

**Figure 6:**
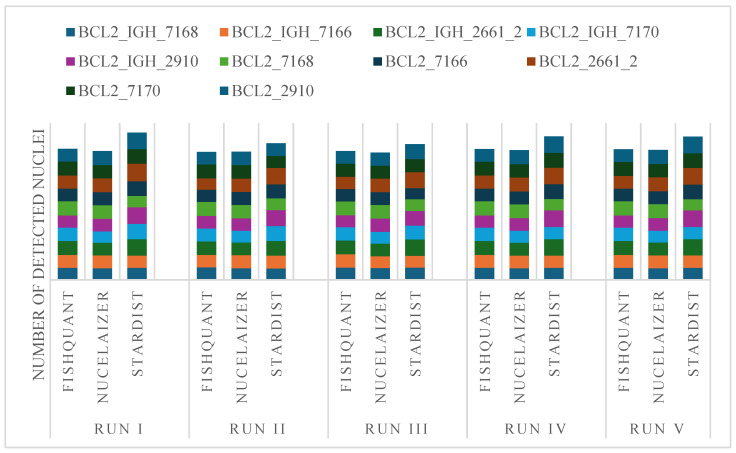
Reproducibility of nuclear segmentation by the tested algorithms in tumor areas labeled using DAPI and marked on digital sections of follicular lymphoma cases.

We also assessed the reproducibility of nuclear segmentation by comparing the performance of the best acting AI algorithms to the adaptive threshold-based results of FISHQuant Figure 6. For each case, we reanalyzed the annotated regions to see which algorithm produced reliable results with the smallest variation between individual runs (Table 2).

To assess the reproducibility and nuclei segmentation variability of the AI-based algorithms, we performed an analysis comparing sample-run variance (VS) and segmented nuclei variance (VP) across two FISH assay types: BCL2/IGH DC DF and BCL2 Break-Apart DC. VS reflected inter-sample consistency, whereas VP indicated intra-sample segmentation stability. For the BCL2/IGH DC DF assay, NucleAIzer exhibited the lowest variance values (VS = 1233.41; VP = 1184.07), suggesting improved reproducibility and segmentation consistency relative to other methods. FISHQuant demonstrated slightly higher but comparable variance (VS = 1339.66; VP = 1286.07), indicative of good overall performance. Conversely, StarDist showed substantially elevated variance metrics (VS = 12,983.58; VP = 12,464.24), highlighting reduced reproducibility. In the BCL2 Break-Apart DC assay, NucleAIzer again showed minimal variance (VS = 559.92; VP = 537.52), confirming its robustness across assay types. FISHQuant displayed moderate variance (VS = 1767.64; VP = 1696.94), reflecting acceptable reproducibility. StarDist presented the highest variance values (VS = 14,021.91; VP = 13,461.03), underscoring significant segmentation inconsistency. These findings highlighted the differential performance of AI-based segmentation algorithms in FISH applications. The adaptive thresholding approach employed by FISHQuant demonstrated balanced performance, particularly in the BCL2 Break-Apart assay (Table 3).

The only 4% differences in VP/VS ratio suggest that FISHQuant, NucleAIzer, and StarDist demonstrated a consistent and comparable performance for both BCL2/IGH DC DF and BCL2 Break-Apart assays with minimal variability between paired measurements, supporting their reliability for quantitative analysis in fluorescence in situ hybridization applications.

### 3.4. Gene Spot Detection Compared to the Detected Nuclei

**Figure 7:**
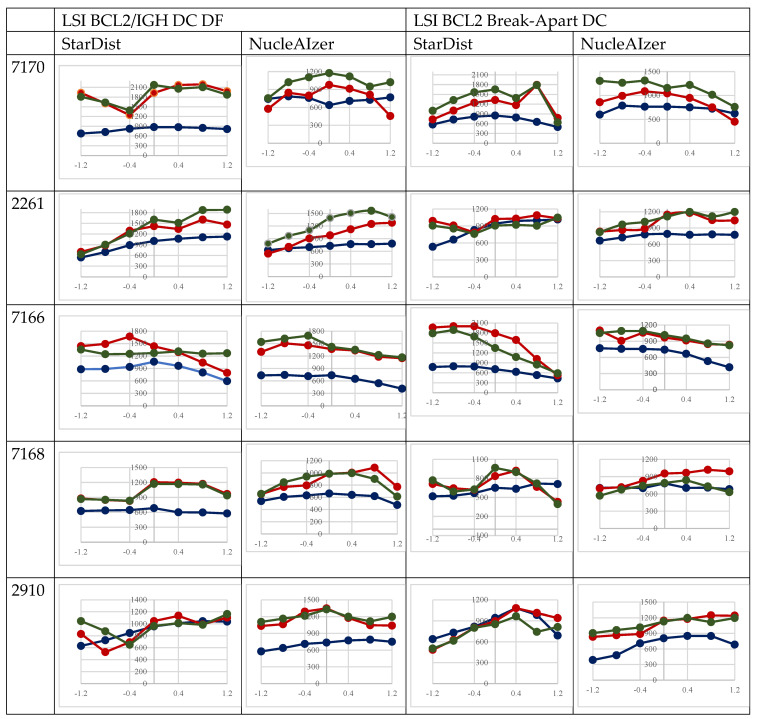
Correlations between the number of FISH gene spots (red and green) and of the segmented cell nuclei revealed through the scanned focus levels in FL FISH samples tested either using samples after a SPEC BCL2 Dual-Color Break-Apart Probe or the SPEC BCL2/IGH Dual-Color Dual-Fusion probe.

The number of diagnostically accepted BCL2 gene-specific fluorescence signals depends on accurately identified nuclei. The spot identifier algorithm we developed is this study detected FITC and TRITC signals across all focal planes exclusively within the greyscale mask images of nuclei delineated by nucleus detection algorithms. As the number of nuclei decreases progressively with increasing distance from the 0th plane in both the upper and lower directions, the number of detectable signals also proportionally diminishes, as seen in Figure 7. The number of gene signals identified per plane on the FITC and TRITC channels demonstrated strong correlations. The StarDist algorithm identified strong correlation between the two channels in both tests (BCL2 Break-Apart: r = 0.894117. BCL2/IGH DC DF: r = 0.92082). At the same time, the NucleAIzer algorithm revealed a strong linear correlation (r = 0.828915) when using the BCL2/IGH DC DF, but only moderate correlation in the case of the BCL2 Break-Apart probe (r = 0.60082).

### 3.5. The Nuclear Segmentation Algorithms in the Genetic Classification of Tumor Cells

To detect the targeted genetic alterations in situ within the FL tissue sections, the green and red fluorescence signals of specific gene loci were identified and rendered to cell nuclei using our dedicated algorithm, followed by using the tested automated nuclear detection methods. Tumor cells were subsequently classified based on the distinct FISH signal pattern of the underlying genetic alterations compared to the normal, which showed either a minimum of three times the spot diameter distance from each other (at fusion probes) or colocalized signal spots (at break-apart probes). To validate the automated classification, manual control classification was conducted on segmented images. Signal detection algorithms were systematically applied to each segmented image, utilizing multiple nuclear segmentation approaches to ensure the robustness and accuracy of the detected genetic signals. Table 4 highlights the comparative genetic analysis of the performance of segmentation methods. We evaluated key metrics, including Recall, Precision, F-measure, and Accuracy of FISH reactions in FL samples using either BCL2 Break-Apart or BCL2_IGH Dual-Color Dual-Fusion probes. The AI-based StarDist and NucleAIzer algorithms showed consistent performance, achieving the highest F-measure and Accuracy scores. These approaches outperformed both the manual counting and the adaptive thresholding-based FISHQuant, especially in Precision and overall classification reliability. While eye-controlled counting demonstrated competitive Recall values, it generally fell short in Accuracy to StarDist and NucleAIzer algorithms, which further supports AI-based solutions as promising tools for improving FISH classification. Across all evaluated cases, the Dice and Jaccard metrics indicate that StarDist and NucleAIzer consistently achieve the strongest overlap with the reference annotations, with values generally ranging from 0.91 to 0.94 for Dice and 0.84–0.89 for Jaccard. These findings suggest that the deep learning-based approaches provide notably more stable and reliable segmentation performance compared with the manual and FISHQuant methods.

## 4. Discussion

The FISH method is still a powerful and widely adopted tool for detecting targeted gene sequences within tissue and cell samples, providing definitive evidence of chromosomal and gene aberrations of diagnostic and therapeutical significance [1,24]. However, the transition from labor-intensive manual microscopy to digital and automated workflows requires precise, reproducible and scalable nucleus detection, and signal localization. These are critical challenges in samples of high cell density with overlapping nuclei, such as in lymphomas. In this study, we evaluated the performance of the adaptive threshold-based FISHQuant and the feely accessible AI-powered Cellpose, NucleAIzer, and StarDist algorithms in both 2D and 3D contexts using FL samples labeled to highlight BCL2 gene rearrangements. The manual, eye-controlled, MarkerCounter annotation mimicking the traditional diagnostic approach served as the reference standard. Our results underscore the advantage of deep learning-based methods over traditional thresholding or manual counting for safe nuclear segmentation. Our results suggest that the NucleAIzer and StarDist algorithms can be offered for upgrading the diagnostic automated FISH analysis software packages [4,7,20,25].

Although several deep learning algorithms for spot detection are available [26,27,28], their integration with nuclear segmentation frameworks remains essential, particularly because only the signals localized within nuclei are diagnostically relevant [14]. The present work focused primarily on nuclear detection and its clinical evaluation, as accurate identification of nuclei is the prerequisite for reliable downstream FISH analysis. While deep neural networks provide multiple options for spot detection, no unified framework is currently accessible that allows simultaneous execution of nuclear and spot detection algorithms in an out-of-the-box manner. To address this limitation, a custom spot detection application has been under development, designed to identify signals specifically within the masks of segmented nuclei across the spot channels [23]. Our intention here was to test contemporary and freely accessible deep learning algorithms if they can improve the nuclear segmentation accuracy of our adaptive thresholding FISHQuant algorithm of diagnostic potential [7,8].

Here we found that all automated methods detected more nuclei than eye-controlled counting. The accuracy of nucleus detection by image processing algorithms is validated by the very similar average number of gene spots per cell nuclei in the AI-based analysis to our assessors findings using eye control (at high kappa values), despite the higher number of cell nuclei detected by AI algorithms (Table 5). The difference in nuclear count is possibly explained by the left-out gene spots we could localize unequivocally to separate cell nuclei. Therefore, AI-based nuclear segmentation may enable pathologists to identify genetic variations across a broader cell population. 

This difference was particularly prominent for the advantage of AI-based approaches in 3D testing. Both NucleAIzer and StarDist consistently recognized and separated more nuclei, especially in crowded FL samples, and maintained this advantage when analyzing multilayer Z-stacks. This 3D potential can be critical for resolving overlapping nuclei and maximizing spatial accuracy. For instance, our multilayer analyses demonstrated that the nuclear counts peaked at the focal plane and diminished as the distance from plane zero increased (see Figure 5). Still, the AI models retained proper signal detection across layers, as evidenced by the robust correlation of FITC and TRITC gene signals per plane (NucleAIzer BCL2/IGH DC DF: r = 0.8289; StarDist BCL2/IGH DC DF: r = 0.9208).

The weak Pearson correlation between Cellpose and StarDist in some cases may result from differences in their segmentation strategies and sensitivity thresholds. Cellpose often adapts better to irregularly shaped or overlapping nuclei, while StarDist relies on star-convex shape modeling that can struggle with such features. As a result, variations in nuclear morphology, staining intensity, or image noise can lead the two methods to produce different nucleus counts, with reduced correlations in those instances [3]. Here, Cellpose clearly underperformed compared to the other algorithms in samples with low or uneven fluorescence signals. Its exclusion from detailed downstream analyses in our study reminds us that general-purpose tools may not be suitable for high-fidelity clinical applications without rigorous optimization [21]. 

Notably, the variance and reproducibility metrics further support these observations. NucleAIzer consistently demonstrated the lowest variance across repeated runs (VS = 1233.41; VP = 1184.07 for BCL2/IGH DC DF; VS = 559.92; VP = 537.52 for BCL2 Break-Apart), which highlighted its robust performance. In contrast, while StarDist achieved slightly higher nuclear detection counts and comparable signal correlation, its run-to-run variance was markedly higher (VS = 12.983.58; VP = 12.464.24 for BCL2/IGH DC DF), indicating some inconsistency in segmentation boundaries. Despite this, StarDist can be very useful for tumor cell classification tasks, achieving high F-measure (0.92–0.94) and Accuracy (0.88) values, which demonstrates that its segmentation quality remains relevant for the downstream genetic analysis. All in all, the object–technology-related differential performance of AI-based segmentation algorithms must be considered in FISH applications. Our observations emphasize the need for algorithm selection that aligns with the unique demands of FISH assays particularly in lymphoma.

From a methodological perspective, the comparative advantages of each algorithm stem from their architectural differences. NucleAIzer utilizes style transfer-based data augmentation and a Mask R-CNN framework with U-Net post-processing for boundary refinement, enabling it to generalize well across diverse staining types and imaging modalities [4]. This domain adaptation is particularly beneficial when variations in sample quality or fluorescence intensities prevail. In contrast, StarDist represents nuclei as star-convex polygons, using radial rays to encode object boundaries, which has proven highly effective in resolving closely clustered or overlapping nuclei [3,13]. These advantages were also confirmed in this study.

The FISHQuant, a traditional adaptive thresholding method, performed moderately with acceptable VP/VS relative agreement (~0.96) and maintained balanced detection in heterogeneous samples. However, its lower Accuracy and F-measure metrics underline its limitations compared to AI approaches. Nevertheless, its ease of implementation, speed, and consistency across runs as well as watershed-based separation still make it a viable tool in settings where computational resources and AI expertise are limited [7].

It must be emphasized that the effectiveness of any segmentation algorithm depends heavily on upstream factors, such as high-quality sample preservation, probe labeling technique, conditions of optimal FISH reaction, image resolution, signal-to-noise ratio, and standardized imaging protocols [8]. Poor sample quality or imaging artifacts can severely impact segmentation accuracy, regardless of the algorithm used. One should also consider fluorophore choice, autofluorescence, clearing chemistry, detector spectra, because, for instance, far-red dyes (e.g., AF-647) consistently yield higher signal-to-noise values than AF-488/594 [29]. Also, FITC may produce autofluorescence in paraffin-embedded samples, which can bias signal detection. Fluorescence whole-slide digitization systems incorporate automatic image enhancement autofluorescence filtering solutions, which in turn affect the visibility of gene signals. In addition, clearing procedures and detector settings can modulate the signal-to-noise ratio and may impact segmentation and spot-calling performance. Routine quality control steps, including normalization, denoising, and artifact removal must therefore be integral to any digital pathology workflow that seeks to take advantage of AI-powered tools [9].

Fluorescence microscopy may also suffer from photon shot noise, camera readout artifacts, and autofluorescence, which impose strict constraints on spatial fidelity and quantitative accuracy. Supervised deep learning methods such as CNNs, U-Nets, and transformer hybrids utilize paired training data to learn strong priors, though they often face challenges in generalization across experiments [30,31]. Self-supervised strategies, including Noise2Noise, Noise2Void, and blind-spot networks, reduce data requirements and improve adaptability by exploiting redundancy without clean targets [32,33,34,35]. Modality-specific AI corrections and hybrid wavelet–deep learning pipelines may further enhance denoising performance, enabling improved signal-to-noise ratio while preserving fine biological structures [36].

The potential limitations of our study include the small case number (five cases tested with two different methods, 10 samples). However, the detected 500–1000 nuclei per sample depending on the method used—on average, 4000 nuclei per each method—should be enough for a valid statistical comparison. Also, there is a lack of alignment to the different methods, since all three third-party applications run the various algorithms, and we aimed to avoid the influence of differing adjustments to each algorithm. However, when implementing any into a complex digital fluorescence FISH evaluation system, the given algorithm must be aligned with the optical path’s capabilities in order to provide the suitable segmentation method that matches the system’s image quality. Further limitations may involve the use of single-center samples, constrained gold standard, no external scanner/probe validation, parameterization incompleteness, potential selection bias, and limited inter-reader analysis (beyond κ).

Finally, the broader clinical implications of using a robust digital image segmentation is of significant importance. Automated FISH analysis, supported by reliable nuclear and gene signal detection, enables large-scale in situ assessment of thousands of cells per sample. This improves the statistical power for detecting rare events, supports the evaluation of tumor heterogeneity—increasingly recognized as a driver of treatment resistance and disease progression [37,38]—and facilitates consistent diagnostic and prognostic classification across laboratories.

Future directions should focus on multi-center and multi-scanner/probe validation, blinded panel ground truth per nucleus, comparisons with additional 3D models, preprocessing ablations, and full code/data re-lease.

In conclusion, our findings show that deep learning-based methods, particularly NucleAIzer and StarDist, may improve the accuracy and reproducibility of cell nuclei segmentation in digital FISH analysis. This can be particularly important in lymphomas of crowded/overlapping tumor cells exemplified by testing for BCL2 gene translocations in FL here. Though the adaptive threshold-based FISHQuant also revealed reasonably good speed and performance consistency, providing useful results [8], the AI-based algorithms in 3D samples more accurately controlled difficult (i.e. with overlapping nuclei) FISH samples. We should emphasize that regular quality control measures can support the consistency of AI methods in detecting gene aberrations for disease classification. A further advantage of automated whole sample FISH analysis that it can offer large-scale detection of genetic tumor heterogeneity of increasing diagnostic and treatment interest [37,38]. The integration of AI-powered NucleAIzer and StarDist image segmentation tools into routine medical practice may enhance diagnostic precision and ultimately improve patient care.

## Figures and Tables

**Table 1:** Pearson correlations of the number of nuclei detected by eye-controlled counting and image analysis algorithms.

Pearson	BCL2/IGH DC DF FISH					BCL2 Break-Apart FISH				
	M.C.	Cellpose	FISHQuant	NucleAIzer	StarDist	M.C.	Cellpose	FISHQuant	NucleAIzer	StarDist
MarkerCounter	1.000	0.415	0.160	−0.839	−0.580	1.000	0.584	−0.199	0.536	0.624
Cellpose	0.415	1.000	−0.102	−0.825	0.034	0.584	1.000	0.490	0.324	0.942
FISHQuant	0.160	−0.102	1.000	−0.133	−0.715	−0.199	0.490	1.000	0.438	0.493
NucleAIzer	−0.839	−0.825	−0.133	1.000	0.335	0.536	0.324	0.438	1.000	0.449
StarDist	−0.580	0.034	−0.715	0.335	1.000	0.624	0.942	0.493	0.449	1.000

**Table 2:** Analysis of the performance robustness of nuclear segmentation algorithms after 5-times repeated analysis using the Pearson correlation matrix in follicular lymphoma FISH samples.

Comparison	FISHQuant	NucleAIzer	StarDist
RUN I vs. RUN II	0.701818387	0.924944594	0.802519762
RUN I vs. RUN III	0.808695753	0.790459051	0.828459987
RUN I vs. RUN IV	0.999969458	0.963942817	0.895763476
RUN I vs. RUN V	0.987741759	0.964726965	0.892947869

**Table 3:** Variance analysis evaluating the reproducibility of nuclear segmentation applications used in annotated areas of follicular lymphomas after FISH tests.

	BCL2/IGH DC DF		BCL2 Break-Apart DC	
Test	VS	VP	VS	VP
FISHQuant	1339.66	1286.07	1767.64	1696.94
NucleAIzer	1233.41	1184.07	559.92	537.52
StarDist	12,983.58	12,464.24	14,021.91	13,461.03

**Table 4:** Summary of the genetic classification abilities of the image analysis methods including Dice and Jaccard matrix in follicular lymphoma samples after FISH-reaction.

	Case Number	Segmentation Method	Recall	Precision	F-Measure	Accuracy	Dice	Jaccard
Break-Apart	2261 BCL2	MarkerCounter. Manual	0.83	0.99	0.90	0.82	0.90	0.82
		FISHQuant	0.77	0.97	0.86	0.75	0.86	0.75
		StarDist	0.92	0.97	0.94	0.89	0.94	0.89
		NucleAIzer	0.92	0.96	0.94	0.89	0.94	0.89
	7170 BCL2	MarkerCounter. Manual	0.84	0.93	0.88	0.79	0.88	0.79
		FISHQuant	0.85	0.94	0.89	0.80	0.89	0.80
		StarDist	0.90	0.92	0.91	0.83	0.91	0.83
		NucleAIzer	0.90	0.93	0.92	0.85	0.92	0.85
	7168 BCL2	MarkerCounter. Manual	0.90	0.75	0.82	0.69	0.82	0.69
		FISHQuant	0.85	0.78	0.81	0.68	0.81	0.68
		StarDist	0.92	0.94	0.93	0.86	0.93	0.86
		NucleAIzer	0.95	0.91	0.93	0.87	0.93	0.87
	7166 BCL2	MarkerCounter. Manual	0.90	0.88	0.89	0.81	0.89	0.81
		FISHQuant	0.88	0.85	0.87	0.76	0.87	0.76
		StarDist	0.92	0.91	0.91	0.84	0.91	0.84
		NucleAIzer	0.93	0.93	0.93	0.87	0.93	0.87
	2910 BCL2	MarkerCounter. Manual	0.87	0.89	0.88	0.78	0.88	0.78
		FISHQuant	0.84	0.86	0.85	0.74	0.85	0.74
		StarDist	0.92	0.91	0.92	0.84	0.92	0.84
		NucleAIzer	0.92	0.94	0.93	0.86	0.93	0.86
Dual-Color Dual-Fusion	2261 BCL2_IGH	MarkerCounter. Manual	0.83	0.88	0.85	0.74	0.85	0.74
		FISHQuant	0.88	0.91	0.90	0.82	0.90	0.82
		StarDist	0.95	0.93	0.94	0.88	0.94	0.88
		NucleAIzer	0.95	0.92	0.94	0.88	0.94	0.88
	7170 BCL2_IGH	MarkerCounter. Manual	0.90	0.85	0.88	0.78	0.88	0.78
		FISHQuant	0.89	0.88	0.88	0.79	0.88	0.79
		StarDist	0.93	0.94	0.93	0.88	0.93	0.88
		NucleAIzer	0.92	0.92	0.92	0.85	0.92	0.85
	7168 BCL2_IGH	MarkerCounter. Manual	0.74	0.83	0.78	0.64	0.78	0.64
		FISHQuant	0.87	0.89	0.88	0.79	0.88	0.79
		StarDist	0.92	0.94	0.93	0.87	0.93	0.87
		NucleAIzer	0.91	0.92	0.91	0.84	0.91	0.84
	7166 BCL2_IGH	MarkerCounter. Manual	0.87	0.89	0.88	0.79	0.88	0.79
		FISHQaunt	0.89	0.88	0.89	0.79	0.89	0.79
		StarDist	0.93	0.95	0.94	0.88	0.94	0.88
		NucleAIzer	0.94	0.91	0.93	0.87	0.93	0.87
	2910 BCL2_IGH	MarkerCounter. Manual	0.85	0.91	0.88	0.78	0.88	0.78
		FISHQuant	0.88	0.88	0.88	0.78	0.88	0.78
		StarDist	0.95	0.93	0.94	0.89	0.94	0.89
		NucleAIzer	0.92	0.93	0.92	0.86	0.92	0.86

**Table 5:** The average spot number in cell nuclei detected by the tested methods.

Sample	Method	Number of Nuclei LSI BCL2/IGH DC DF	Number of Spots FITC	Number of Spots TRIC	Number of Nuclei LSI BCL2 Break-Apart DC	Number of Spot FITC	Number of Spots TRIC
BCL2 7166	Manual Quantification	435	1479	1530	789	2761	2781
	NucleAIzer	737	2505	2556	743	2600	2632
	StarDist	709	2410	2440	843	2950	2954
BCL2 7170	Manual Quantification	433	1472	1511	640	2240	2275
	NucleAIzer	638	2169	2173	765	2677	2699
	StarDist	873	2968	2977	843	2950	2931
BCL2 2910	Manual Quantification	491	1670	1677	487	1704	1711
	NucleAIzer	732	2488	2433	807	2824	2899
	StarDist	957	3253	3221	940	3290	3310
BCL 27168	Manual Quantification	412	1400	1430	608	2128	2201
	NucleAIzer	663	2254	2310	781	2733	2760
	StarDist	685	2352	2329	651	2267	2278
BCL2 2661	Manual Quantification	429	1458	1421	334	1169	1210
	NucleAIzer	735	2499	2514	792	2762	2722
	StarDist	942	3199	3202	1004	3544	3514
Average		658.1	2238.4	2248.3	735.1	2573.3	2591.8
Average spot/Nuclei			3.4	3.41		3.5	3.525

## Data Availability

Data supporting reported results are available on reasonable request from the corresponding author.
